# Metapopulation modelling of long-term urban habitat-loss scenarios

**DOI:** 10.1007/s10980-017-0504-0

**Published:** 2017-03-25

**Authors:** Laura J. Graham, Roy H. Haines-Young, Richard Field

**Affiliations:** 1 Geography and Environment, University of Southampton, Southampton, SO17 1BJ UK; 2 School of Geography, Sir Clive Granger Building, University of Nottingham, Nottingham, NG7 2RD UK

**Keywords:** Urban ecology, Landscape-scale, Metapopulation modelling, Scenarios, Incidence function model, Multiple criteria decision analysis, Birds, Amphibians

## Abstract

**Context:**

Increasing human populations in urban areas pose a threat to species’ persistence through habitat loss and fragmentation. It is therefore essential that we develop methods to investigate critical habitat loss thresholds and least detrimental landscape configurations.

**Objectives:**

We develop a framework to assess how the pattern of habitat loss impacts the ecological and social characteristics of a landscape and how this varies depending on the species and criteria by which it is judged.

**Methods:**

We use a scenario-based approach to test six propositions in which habitat is lost preferentially based on patch characteristics. We use eight bird and two amphibian species as indicator species. To compare scenarios, we present a method combining the output from a metapopulation model with measures of social impacts of land-cover change in a multiple criteria decision analysis. We also determine whether a habitat loss threshold exists, below which small loss of habitat can lead to large loss of species’ occupancy.

**Results:**

We found that, of the scenarios presented, preferentially losing common habitats and smaller patches was least detrimental for both ecological and social factors. Threshold effects were found for all but the generalist bird species.

**Conclusions:**

We have outlined a workflow which allows for transparent, repeatable comparison between landscapes. This workflow can be used to compare urban landscape plans, or to develop general understanding of the impacts of different forms of habitat loss. Reassuringly, the recommendations based on the scenarios presented are in keeping with received conservation wisdom: to prioritise larger and/or rarer patches.

**Electronic supplementary material:**

The online version of this article (doi:10.1007/s10980-017-0504-0) contains supplementary material, which is available to authorized users.

## Introduction

Protecting urban biodiversity can have benefits for conservation at broader scales. Urban green and semi-natural spaces can provide habitat for native species that are under threat from agricultural intensification of the wider countryside (Tratalos et al. 2007). This is in part due to the heterogeneity of remnant habitats within urban areas (McKinney 2008). Urban remnant habitat has been found to be important for bird (Gregory and Baillie 1998; Mo et al. 2000) and invertebrate taxa (Angold et al. 2006; Soga et al. 2014; Baldock et al. 2015). Urban habitat patches can also have the effect of increasing the connectivity of the wider landscape, in effect acting as ‘stepping stones’ (Fischer and Lindenmayer 2002; Dearborn and Kark 2010). Conversely, the loss of a critical patch within the network can impair the connectivity of the landscape (Jordán et al. 2003). Conserving urban biodiversity can also have benefits for conservation by creating a positive feedback loop: if people are reconnected with nature, they are more likely to support conservation initiatives (Miller 2005). For example, children who experience the natural world first hand are more likely to become passionate about conservation than those that do not (Chawla 1999). Personal experience with nature can also shape values in adults; Dearborn and Kark (2010) argued that if policy makers have day-to-day experience of urban nature, this will have a positive impact on conservation policy.

The arguments above assume support for a conservation case for nature. It is important also to consider the wider benefits that urban conservation can have for human well-being. Positive associations have been found between green space and physical health (Nielsen and Hansen 2007; Hartig et al. 2014), mental health (Annerstedt et al. 2012; Alcock et al. 2014) and crime reduction (Troy et al. 2012; Wolfe and Mennis 2012). Although most studies tend to report positive associations between green space and human well-being, positive relationships with species richness have also been found (Fuller et al. 2007; Carrus et al. 2015). Natural spaces have also been found to be associated with improved economic success of a region. For example, many studies have shown a positive relationship between green spaces and house prices (Garrod and Willis 1992; Gibbons et al. 2014). City planners should therefore consider ecological, economic and social criteria in decision making (Wu 2009).

Ecological factors that planners need to take account of include habitat loss and fragmentation, which pose a significant threat to species’ persistence (Tilman et al. 1994), with the effects of land-use change being potentially more significant than other major threats, such as climate change and the introduction of invasive species (Haines-Young 2009). Human population change is recognised as one of the main drivers of land-use change (Foresight Land Use Futures Project 2010). At present, 54% of the global population live in urban areas, and the urban population is expected to increase by around 1.5–2% per year to accommodate increasing populations (World Health Organisation 2015). As a result, further habitat loss in urban areas is inevitable, and methods to investigate critical habitat loss thresholds and landscape configurations which pose the least threat to biodiversity are essential (Lin and Fuller 2013).

The concept of ecological thresholds is important to consider when assessing the impact of an external perturbation such as climate change, overexploitation, introduction of invasive species, or in this case habitat loss (Andersen et al. 2009). The impacts of habitat loss on species’ persistence are not necessarily linear (Swift and Hannon 2010), and it is possible that a ‘critical threshold’ of habitat loss exists, below which there can be abrupt changes in populations. Thresholds such as these exist in many ecological systems and can be a useful way to establish a minimum viable habitat size (Walker and Meyers 2004). As such, it is important to develop methods to identify critical habitat loss and to ensure that in land-use planning the coverage of habitat does not decrease below these thresholds.

Here we investigate optimal landscape configuration by testing a set of habitat loss propositions. A habitat patch’s vulnerability to destruction can depend on characteristics such as patch geometry, type and spatial location. First, developments can act as contagions in the landscape (Laurance 2008), and thus patches that are closer to existing developments are more likely to be lost. Second, the size of a habitat patch may affect its vulnerability. For example, disproportionately large losses of small habitat patches have been found in forest habitat (Altamirano et al. 2013). A possible reason for this is that species in small patches have a higher probability of extinction (Bennett and Saunders 2010), and therefore the destruction of smaller patches is considered less detrimental to the entire landscape. Conversely, urban areas tend to be characterised by small habitat patches (Di Giulio et al. 2009), so urbanisation may put larger habitat patches at risk, either through loss of these larger patches or a reduction in size through fragmentation. Third, habitat type can determine the vulnerability of a patch. For example, in the UK, the Biodiversity Offsetting Green Paper outlines a metric that assigns a ‘distinctiveness’ to any habitat under threat of development (Defra 2013). The green paper suggests that the government may want to promote development on patches with low distinctiveness. Within the UK context, this means that habitats such as semi-improved grassland are more likely to be lost than woodland habitats.

In this paper we present a workflow which allows for transparent, repeatable comparison of outcomes (particularly conservation and social outcomes) between landscapes resulting from different land-use policies. We use a scenario-based approach to test six propositions in which habitat is lost preferentially based on patch characteristics such as those outlined above: small patches lost first, large patches lost first, nationally common habitats lost first, locally common habitats lost first, habitat loss radiates from most recent developments, patch shrinkage. In doing so we address the following questions: (1) How does the pattern of habitat loss impact the ecological and social characteristics of a landscape and what does this mean for sustainability? (2) How does the answer to (1) vary depending on the species and the criteria by which it is judged? (3) Does a habitat loss threshold exist and how does this vary between species and habitat loss pattern? We expect the generalist species to be more affected by the size of habitat patches lost rather than their type, the farmland specialists to fare better in the contagion scenario because this is likely to leave more farmland intact, and the woodland specialists to be adversely affected by all scenarios, but the scenarios which conserve rarer habitats (e.g. woodland) will have the least impact.

## Methods

### Overview of methods

**Fig. 1:**
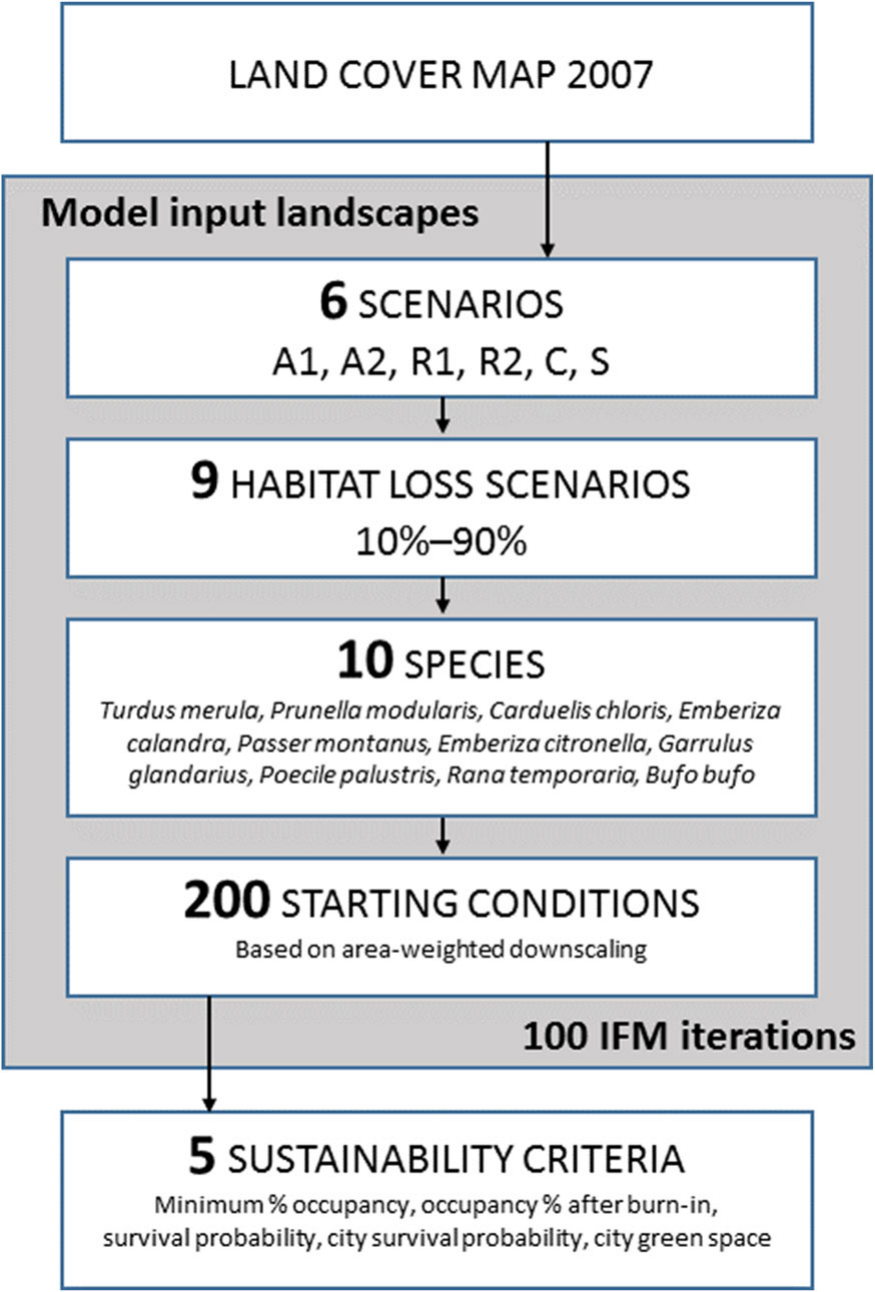
Diagram showing the full study design

As illustrated in Fig. 1, we first manipulated semi-natural land cover in the study area (Nottingham City, UK) to create six groups of scenarios, each with 9 landscapes with 10–90% semi-natural cover loss. Second, we downscaled 2 × 2 km species observations for 10 species to patch level, creating 200 starting conditions of occupancy for each species. Third, we simulated species occupancy for each of the 54 landscapes and 200 starting conditions using 100 replicates of the incidence function model (IFM). Fourth, from the output of the IFM simulations we calculated five measures of landscape-scale sustainability (three represented ecological sustainability, and two social sustainability). Finally, we used multiple-criteria decision analysis (MCDA) to compare the 6 scenarios, where each unique combination of landscape (n = 9), species (n = 10), starting condition (n = 200) and sustainability measure (n = 5) was a ‘criterion’ in the analysis (n = 90,000 criteria, Fig. 1).

### Study area

**Fig. 2:**
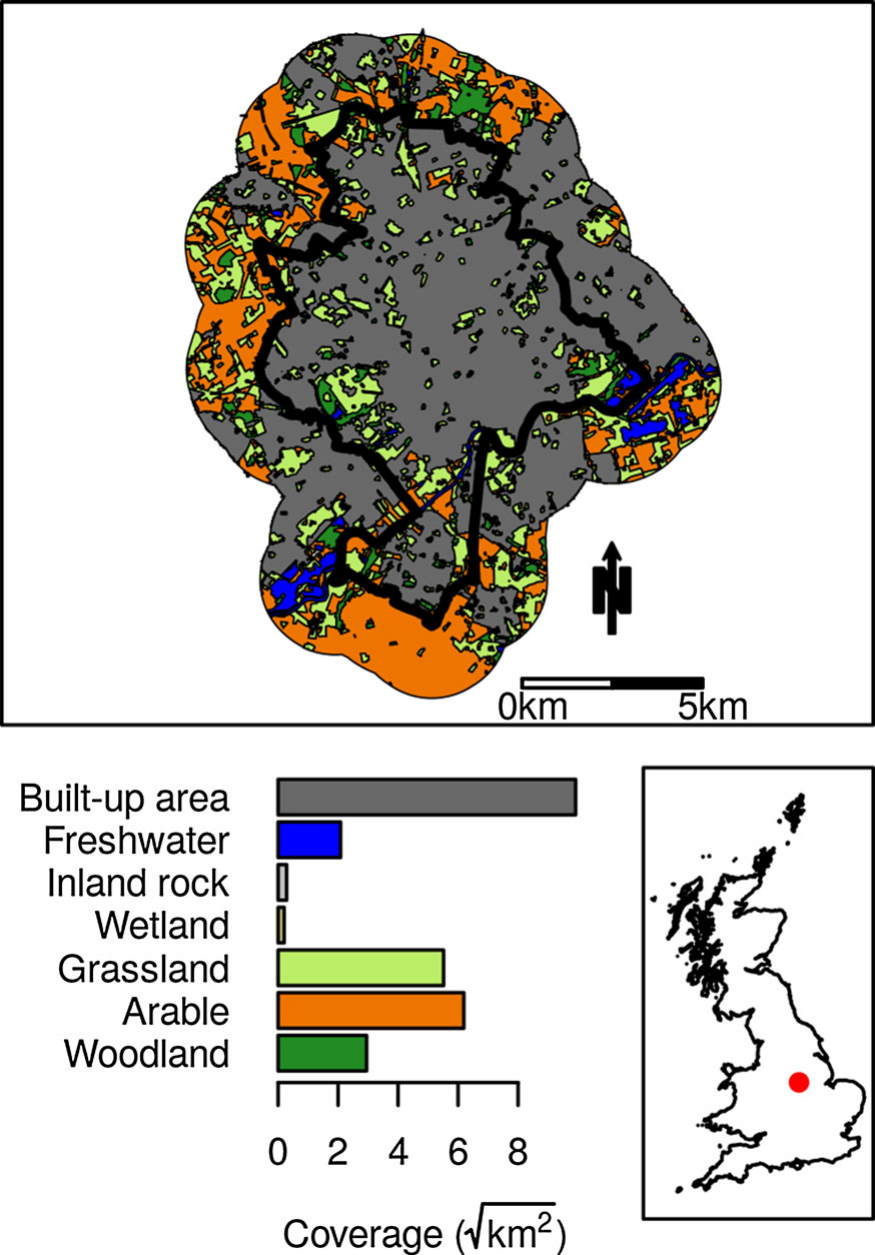
Study site of Nottingham with a 2 km buffer showing location and coverage of grouped land cover types. Inset map shows location within the Great Britain

We used the case study site of Nottingham City unitary authority, with a 2 km external buffer, as a starting point from which to develop habitat-loss scenarios (Fig. 2). Nottingham is a typical medium-to-large urban area located in the East Midlands, UK. This represents a case study area that captures a situation typical of those facing many European cities. In the UK, the unitary authority is the level at which planning decisions are made; the buffer was included to allow for dispersal from outside the study (‘decision making’) area.

### Landscape scenarios

Scenarios can be an effective way to understand the consequences of decisions, and are therefore commonly used in planning (Ash et al. 2010). To investigate the ways in which the type, size and configuration of habitat loss affect landscape-scale sustainability, we created six scenario groups of potential future fragmentation patterns (Table 1). For each scenario group, we created 9 land cover maps where habitat cover was reduced by 10% of the present total habitat cover, then 20% and so on until 90% of the present-day habitat cover was lost; this resulted in 54 different scenario landscapes. We used Land Cover Map (LCM) 2007 (Morton et al. 2011) as the base data for scenario creation. This is a remotely sensed dataset which details the types of land cover within the landscape. We modified these data based on sets of rules, as described below, to create the scenario landscapes. Habitat cover was defined as those classes of the LCM 2007 data associated with the focal species, excluding freshwater (the classes are broadleaved woodland; coniferous woodland; arable and horticulture; improved grassland; rough grassland; neutral grassland; acid grassland; fen, marsh and swamp; heather and heather grassland). The freshwater category of the LCM 2007 data mainly includes major water bodies, and therefore the loss of these ‘patches’ is not realistic.

**Table 1:** Description of the six scenario groups and the methods used to create each of the landscapes in these groups

Scenarios based on patch area (A)	
Scenario Group A1. Smallest patches lost first. (Altamirano et al. 2013)	A1.1 Sort patches into ascending order of area A1.2 Calculate cumulative area A1.3 Remove first n patches whose area equals 10% of total habitat area A1.4 Save resulting landscape A1.5 Repeat steps 3–4 for all percentage classes until 10% original habitat area remains
Scenario Group A2. Largest patches lost first. (Di Giulio et al. 2009)	A2.1 Sort patches into descending order of area A2.2 Calculate cumulative area A2.3 Remove first n patches whose area equals 10% of total habitat area A2.4 Save resulting landscape A2.5 Repeat steps 3–4 for all percentage classes until 10% original habitat area remains
Scenarios based on rarity of habitat type (R)	
Scenario Group R1. Habitat loss in order of national rarity. (Defra 2013)	R1.1 Calculate order of national habitat rarity (1 being most common) R1.2 Sort patches into ascending order of habitat rarity (order within rarity classes random) R1.3 Calculate cumulative area R1.4 Remove first n patches whose area equals 10% of total habitat area R1.5 Save resulting landscape R1.6 Repeat steps 4–5 for all percentage classes until 10% original habitat area remains
Habitat loss in order of local rarity. (Defra 2013)	R2.1 Calculate order of local habitat rarity (1 being most common) R2.2 Sort patches into ascending order of habitat rarity R2.3 Calculate cumulative area R2.4 Remove first n patches whose area equals 10% of total habitat area R2.5 Save resulting landscape R2.6 Repeat steps 1–5 for all percentage classes until 10% original habitat area remains
Scenarios based on proposed developments acting as contagion (C)	
Scenario Group C. Developments act as a contagion on the landscape. (Laurance 2008)	C.1. Create ‘developments’ layer from Nottingham City Local Plan C.2. Calculate distance to nearest development for each patch C.3. Sort patches into ascending order of distance to nearest development C.4. Calculate cumulative area C.5. Remove first n patches whose area equals 10% of total habitat area C.6. Save resulting landscape C.7. Repeat steps 1–6 for all percentage classes until 10% original habitat area remains—updating the developments layer to include removed patches
Scenarios based on habitat shrinkage (S)	
Scenario Group S. Habitat fragmentation approximated by patch shrinkage. (Di Giulio et al. 2009)	S.1. Create −10% buffer using `Buffer by percentage’ in QGIS 2.4.0 S.2. Save resulting landscape S.3. Repeat steps 1–2 for all percentage classes (e.g. buffer = −20, −30% etc.)

For each scenario, we defined sets of rules for modifying the land cover of the study site based on patterns of habitat loss seen in the ecological literature (Table 1). The modification rules for scenarios A1 and A2 work on the assumption that habitat loss is dependent on the area of the patch. A1 is based on the idea that small habitat patches are more vulnerable to development than large patches. Here, we sorted the patches into ascending order of patch area, calculated the cumulative area, and removed the appropriate percentage of total habitat area for each percentage class, starting with the smallest patches. For example, for the scenario of loss of 10% of present total habitat cover, patches were removed in ascending order of size until the 10% habitat loss had been achieved. In contrast, in A2 the largest patches were removed first. Here, the patches were sorted into descending order of patch area, while the other steps were the same as for A1.

Scenarios R1 and R2 were created to investigate how the type of habitat lost affects species’ persistence. Here the assumption is that habitat loss is based on the distinctiveness, or rarity (hence the label R) of the habitat patch, and that the most common habitats are likely to be more vulnerable to development—for example under the policy recommendation from the biodiversity offsetting Green Paper (Defra 2013). For scenario R1, we created a measure of national rarity by calculating the total coverage of each habitat class in the UK from the LCM 2007 data (Table S1a) and applying a value from 1 to 10 for each of the ten habitat classes where 1 is the most common. We removed patches of each rarity class from the most to the least common habitat type until no more of that class remained (random order within rarity classes), we calculated the cumulative area, and created shape files containing the appropriate amount of habitat cover for each percentage class from the land cover mosaic. For scenario R2 a modified approach was taken to account for the fact that, locally, the habitat could be managed adaptively. Here, the rarity measure was calculated in the same way, but the LCM 2007 data were limited to the study site and, after each 10% loss of habitat, the rarity measure was recalculated to reflect the new composition of habitat types. The measure of local rarity for the present day is shown in Table S1b.

Scenario group C is based on the idea that development acts as a contagion in the land cover mosaic and that habitat destruction tends to be spatially clustered. We based the new developments on those proposed for Nottingham City in the current Local Plan. We obtained maps of the proposed Nottingham Local Plan from Nottingham City Council (2005a, b). We imported these maps into ArcMap 10.0, georeferenced to match the LCM 2007 data, and created a new ‘developments’ layer by digitising all proposed development sites. The attribute table for the shape file was ordered by distance to the nearest development, and a cumulative area of patches calculated. For each of the nine percentage classes, we removed the appropriate amount of habitat.

Scenario group S was created to investigate the impact of patch shrinkage and fragmentation. The LCM data were first merged by LCM habitat class to remove the ownership boundaries. For each percentage class, we reduced the patches in size by the appropriate amount. To do this we used the ‘Buffer by percentage’ tool in QGIS 2.4.0 setting a 90% buffer for 10% habitat loss, 80% buffer for 20% habitat loss, and so on.

### Species data

To investigate the impacts of habitat loss across a suite of indicator species, we selected species with a range of habitat specialisms and dispersal abilities (see Table 2 for full details). Occurrence records for eight bird species (*Turdus merula, Prunella modularis, Carduelis chloris, Emberiza calandra, Passer montanus, Emberiza citrinella, Garrulus glandarius, Poecile palustris*) were provided by Nottinghamshire Birdwatchers. Occurrence records for two amphibian species (*Rana temporaria, Bufo bufo*) were downloaded from the National Biodiversity Network Gateway (National Biodiversity Network 2014) using the ‘rnbn’ package (Ball and August 2013). The bird species are generalists (*T. merula, P. modularis, C. chloris*), farmland specialists (*E. calandra, P. montanus, E. citrinella*) and woodland specialists (*G. glandarius, P. palustris*). Information on species–habitat associations was taken from Wernham et al. (2002) and Holden and Cleeves (2006) for birds, and from Beebee and Griffiths (2000) for amphibians. Dispersal distances for birds mainly came from Paradis et al. (1998); they provide both breeding and natal distances, and we used the natal distances. We obtained dispersal distances for additional species from Wernham et al. (2002) (*Emberiza calandra*), Broughton et al. (2010) (*Poecile palustris*) and Gilioli et al. (2008) (*Rana temporaria, Bufo bufo*).

**Table 2:** Broad habitat type (based on Land Cover Map [LCM] 2007), mean natal dispersal distance and minimum habitat requirement for each species

Species	Common name	Dispersal (km)	LCM class	Min. area (ha)
*Turdus merula* (L.)	Blackbird	3.300	1, 2, 3, 4, 5, 6, 7, 8	0.02
*Prunella modularis* (L.)	Dunnock	2.100	1, 2, 3, 4, 5, 6, 7, 8	0.02
*Carduelis chloris* (L.)	Greenfinch	4.200	1, 2, 3	0.25
*Emberiza calandra* (L.)	Corn bunting	4.000	3, 4, 5, 6, 8	2.50
*Passer montanus* (L.)	Tree sparrow	8.000	1, 2, 3	0.12
*Emberiza citrinella* (L.)	Yellowhammer	8.400	3, 5, 10, 11	0.03
*Garrulus glandarius* (L.)	Jay	3.500	1, 2	0.32
*Poecile palustris* (L.)	Marsh tit	0.885	1	2.10
*Rana temporaria* (L.)	Common frog	1.000	1, 2, 3, 4, 5, 6, 8, 9, 16	0.02
*Bufo bufo* (L.)	Common toad	0.700	1, 2, 5, 6, 8, 9, 16	0.02

LCM classes: 1—Broadleaved woodland, 2—Coniferous woodland, 3—Arable and horticulture, 4—Improved grassland, 5—Rough grassland, 6—Neutral grassland, 8—Acid grassland, 9—Fen, marsh and swamp, 10—Heather, 11—Heather grassland, 16—Freshwater. Number of LCM classes approximates generalism

We created habitat maps for the individual species by modifying each of the scenario landscapes (described in the previous section) to reflect the species–habitat associations and minimum patch size requirements (see Table 2). We dissolved the boundaries created by land ownership, demarcations between habitat types, and paths and small roads (≤3 m in width) to make patches realistic in terms of how the species use them.

The IFM requires patch-level occupancy information, but the data available are at 2 × 2 km grid cell level. We employed the area-weighted downscaling technique described in Graham et al. (2015) to create a set of 200 species patch occupancy configurations, hereafter called starting conditions. The method takes as an input species’ presence data at the 2 × 2 km grid cell level and downscales to individual patches based on the patch area. Within an occupied 2 × 2 km grid cell, patches are randomly allocated as occupied by a species with a weighting towards larger patches. The proportion of patches assigned as occupied is equal to the proportion of grid cells occupied in the total landscape. This assumes species occupancy is self-similar regardless of scale. The method employed is stochastic, and therefore a level of uncertainty is present. To incorporate this uncertainty into the analysis we created 200 starting conditions for each species.

### Model simulation

We use the IFM to estimate species’ occupancies under the landscape scenarios. The IFM is a stochastic patch occupancy model which simulates species’ extinction and colonisation within habitat patches across a specified time period. Species’ extinctions are modelled with a probability that is a function of patch area, while colonisations are modelled with a probability that is a function of patch isolation (Hanski 1994).

We estimated the parameters for the IFM by fitting eight years of species-occupancy data for each of the 200 starting conditions for the present-day landscape using a logistic regression model with patch area and connectivity as independent variables. This resulted in a set of 200 parameter combinations. We simulated species’ patch occupancies using the IFM in R v3.0.2 (R Core Team 2015) for each of the ten species, 200 parameter combinations and 54 landscape scenarios. Each was simulated for 500 timesteps and 100 replicates. The simulations were run on the University of Nottingham’s High Performance Cluster (Intel Sandybridge E5-2670 2.6 GHz, 20 GB RAM allocated). We used the classic formulation of the IFM explained in detail by Hanski (1994, 1999). Full code and data for the simulations are available on GitHub (https://github.com/laurajanegraham/ifm_r).

From the output of IFM simulations, we defined four measures landscape-scale sustainability. Minimum occupancy %, occupancy % after burn-in (175 time steps) and survival probability represent ecological sustainability. The burn-in period chosen was the point at which most species’ occupancies were stable for the current landscape configuration. Species’ survival probability within the unitary authority (‘city survival probability’) represents ‘social sustainability’ as a measure of the existence value of biodiversity for people. A further measure of social sustainability was derived from the land-cover data: total amount of natural space inside the unitary authority which represents access to green space (‘city green space’). Although we simulate species’ occupancy across a number of time steps, the output should not be viewed as an explicit prediction at a particular point in time, but rather an indicator of the stability of the metapopulation in that particular landscape configuration.

### Ranking scenarios

Alternative land management scenarios need to be judged by many criteria; often a scenario which is rated highly on one set of criteria may be rated poorly on another. For example, in our case study, we may find that management scenarios that are beneficial for woodland species are not beneficial for farmland species. In urban planning, it is also essential to be able to integrate additional non-ecological criteria into the analysis. Ranking scenarios can be considered a ‘multiple criteria decision analysis (MCDA) problem’: a problem where multiple, often conflicting, criteria are balanced to find an optimal solution. We approached the scenario comparison as an MCDA problem where the scenarios are the actions and the results from model simulation under each parameter set are the criteria. This means that the uncertainty in the parameter estimates is taken into account in the analysis.

We used the PROMETHEE method for MCDA [see Brans et al. (1986) for a description of the full method]. PROMETHEE is an outranking method that allows pairwise comparison of actions (in this case, the scenarios) for a finite set of criteria. A multi-criteria matrix is calculated with criteria as the rows and actions as the columns (Table 3a).

**Table 3:**
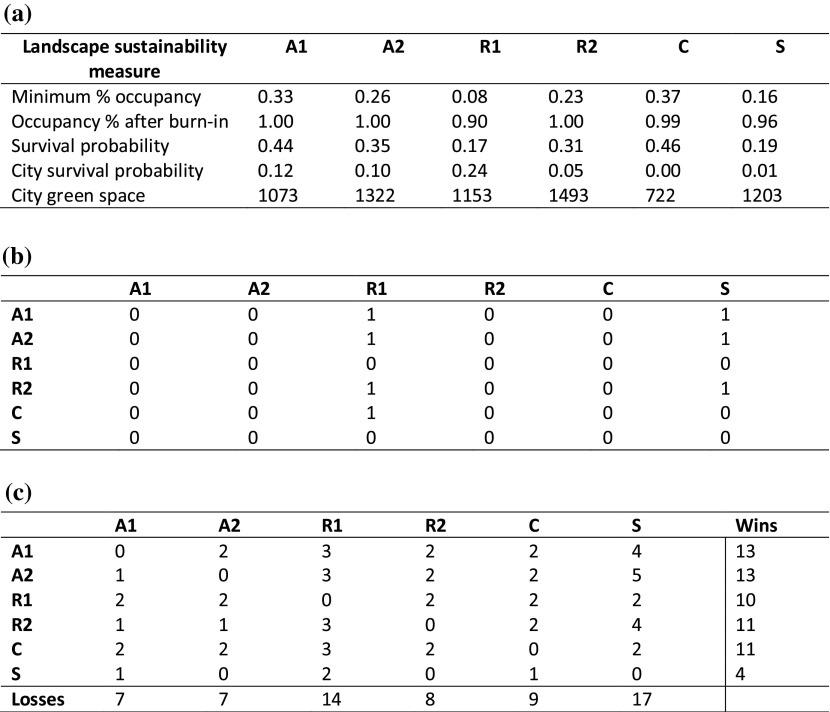
Worked example of the PROMETHEE method for *Emberiza calandra* for 30% habitat loss and the first parameter set. First the multiple criteria matrix is calculated (**a**) where the columns represent the scenarios (*A1*—smallest patches lost first; A2—largest patches lost first; R1—nationally common patches lost first; R2—locally common patches lost first; C—developments as contagion; S—patch shrinkage); then the partial preference matrix is calculated for each criteria (**b** shows the matrix for survival probability); finally the total preference matrix is calculated (**c**)

For each criterion, a partial preference matrix is created; this is a record of all pairwise comparisons between scenarios. Here, the action that maximises (or minimises) the given criterion gains a point, and if there is no significant difference between scenarios no point is awarded. For each comparison we compared means of the output from each simulation using a Mann–Whitney U test and defined a significant difference between two scenarios as that where P < 0.05. The partial preference matrix for the survival probability row for a worked example is given in Table 3b.

These partial preference matrices can be summed to create a total preference matrix for all criteria and the scenarios are ranked according to the total points awarded. The rankings can be gained by using either the total number of times a scenario has won or the total number of times a scenario has lost. If the preference orders gained are identical, then the solution is robust. It is possible for the preference orders to differ, and in this case it may be that two scenarios are incomparable, meaning that neither scenario can be judged to be better than the other based on the information. Table 3c shows the total preference matrix for the worked example. Here it can be seen that the preference orders are almost identical regardless of whether calculated from the ‘wins’ or the ‘losses’, meaning the solution is fairly robust. In this case, R2 and C are equal when judged by ‘wins’, and R2 is better using ‘losses’.

For each scenario, a ‘criterion’ was defined as the unique combination of the five sustainability measures, the nine habitat cover percentage classes, 10 species and 200 starting conditions, resulting in 90,000 criteria for comparison.

### Habitat loss threshold

From the results of the simulations, we created a data set providing the mean % of suitable habitat area occupied for each species and scenario at the different habitat loss percentage classes after the burn-in period of t = 175 time steps. The mean was taken across the 200 starting conditions and 100 model iterations. From this data set we identified whether a habitat threshold exists for each species and scenario. We defined a threshold as the first instance (if any) where the decrease in species’ occupancy between habitat loss percentage classes (which differ by 10% of present-day habitat area) is greater than 30% of the species’ present-day occupancy. This represents a disproportionate amount of species loss for the associated loss of present day habitat.

## Results

### Model simulation

Model simulations took a mean time of 2.3 h (minimum time 0.07 h for *Poecile palustris* scenario S 90% habitat loss, maximum time 53.6 h for *Rana temporaria* scenario S 10% habitat loss). Simulation was not possible for *Bufo bufo* for A1 90% loss and C 60–90% loss or *Poecile palustris* for A1 80–90% loss, R2 80–90% loss and C 50–90% loss. For these scenarios, there was no remaining habitat in grid cells where the species have been recorded, and thus the species’ occupancies were recorded as zero. The means and standard deviations for the model parameters used are given in Table S2.

### Scenario comparison

**Fig. 3:**
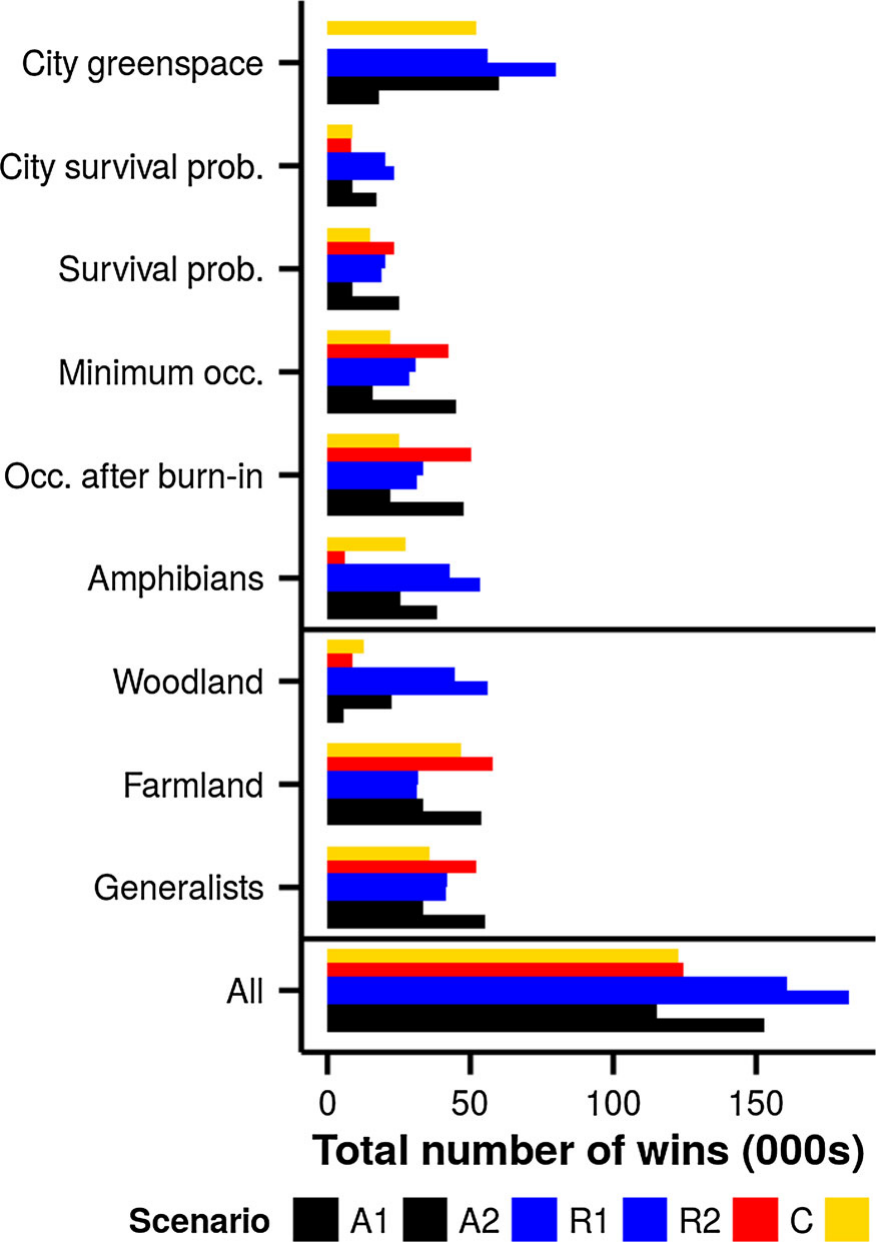
Results of the PROMETHEE comparison. Pairwise comparisons between each scenario for all 200 parameter sets. *Horizontal line* separates all results, results grouped by species’ habitat specialism and results grouped by sustainability measure. *Colours* identify scenario groups (see Table 1). (Color figure online)

The overall comparison of all species using all landscape-scale sustainability measures suggests that the highest-ranked landscape scenario is R1, where the most common habitats nationally are lost first. This is closely followed by R2, locally common habitats lost first, and A1, where the smaller patches are lost first. The full results are shown in Fig. 3. For the four highest-ranked scenarios (R1, R2, A1 and C) the ordering achieved is equal whether judged by wins or losses.

**Fig. 4:**
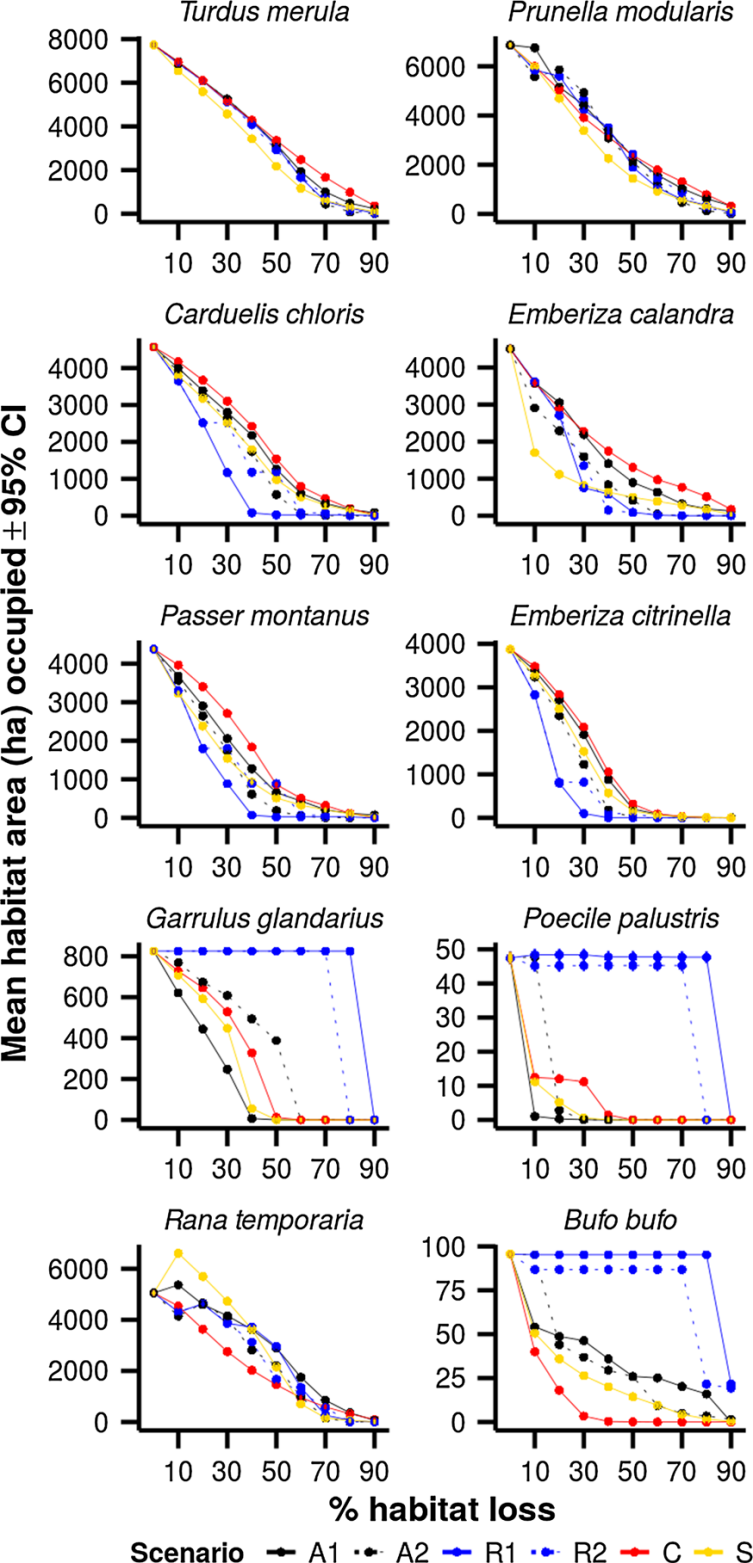
Simulated occupancies by species at timestep t = 175 for each scenario under increasing habitat loss. *Colours* identify scenario groups (see Table 1). NB. 95% Confidence intervals have been plotted but are too narrow to be visible. (Color figure online)

To investigate whether there was a difference depending on species’ traits, we grouped the species into their habitat specialisms before calculating the total preference matrix (Fig. 3). These are: generalists (*Turdus merula*, *Prunella modularis*, *Carduelis chloris*), farmland specialists (*Embriza calandra*, *Passer montanus*, *Embriza citrinella*), woodland specialists (*Garrulus glandarius*, *Poecile palustris*) and amphibians (*Rana temporaria*, *Bufo bufo*). For woodland and amphibian species, the same two scenarios (R1 and R2) were highest ranking. For amphibian species scenario A1 was next highest ranked. It should be noted that the amphibians can be further divided into a generalist and a specialist (*Rana temporaria* and *Bufo bufo* respectively), and when considering the analysis at species level *R. temporaria* was least impacted by scenario A1, and *B. bufo* was least impacted by R1 (this can be seen in Fig. 4). For generalists and farmland species however, scenarios A1 and C were overall highest ranking.

Five measures of ecological and social sustainability were used to calculate the relative performance of the scenarios. To check whether the order was robust to the measurement used, and thus whether the specific measurement used is important, the analysis was broken down by sustainability measure (see Fig. 3). When considering the analysis based only on the ecological sustainability measures (minimum occupancy %, occupancy % after burn-in and survival probability), scenarios A1 and C were highest ranking when the results for all species were combined. For the social sustainability measures (city survival probability and city green space), however, the scenarios where the rarer habitats are conserved (R1 and R2) were highest ranking. For provision of green space within the city, scenario A2 (smallest patches retained) also had a less detrimental effect than the remaining scenarios.

### Habitat loss threshold

The occupancy at t = 175 was calculated for each species and scenario landscape and plotted in Fig. 4. Table 4 shows the points at which a habitat loss threshold was detected. Thresholds were detected for all scenarios for *Poecile palustris* and *Bufo bufo*, for all but scenario A1 for *Garralus glandarius* and for some scenarios for *Emberiza calandra*, *Passer montanus*, *Emberiza citrinella* and *Rana temporaria*. It should be noted that the thresholds were very variable, from some at 10% habitat loss, to some at 90% habitat loss; this occurred even within species (e.g. *Poecile palustris*).

**Table 4:** Thresholds for those species and scenario combinations which display such an effect. Species–scenario combinations not present in the table did not display a threshold effect

Species	Scenario	Habitat loss threshold (%)	Decrease in mean occupancy
*Emberiza calandra*	A2	0–10	35.38
*Emberiza calandra*	R1	20–30	43.41
*Emberiza calandra*	R2	20–30	30.32
*Emberiza calandra*	S	0–10	62.18
*Passer montanus*	R1	10–20	34.28
*Passer montanus*	R2	10–20	34.05
*Emberiza citrinella*	R1	10–20	52.45
*Emberiza citrinella*	R2	10–20	51.79
*Garrulus glandarius*	A2	50–60	47.01
*Garrulus glandarius*	R1	80–90	99.99
*Garrulus glandarius*	R2	70–80	100.00
*Garrulus glandarius*	C	40–50	38.20
*Garrulus glandarius*	S	30–40	47.61
*Poecile palustris*	A1	0–10	97.78
*Poecile palustris*	A2	10–20	94.74
*Poecile palustris*	R1	80–90	100.00
*Poecile palustris*	R2	70–80	95.31
*Poecile palustris*	C	0–10	73.76
*Poecile palustris*	S	0–10	76.38
*Rana temporaria*	R1	50–60	31.71
*Bufo bufo*	A1	0–10	43.29
*Bufo bufo*	A2	10–20	53.57
*Bufo bufo*	R1	80–90	76.98
*Bufo bufo*	R2	70–80	68.32
*Bufo bufo*	C	0–10	58.12
*Bufo bufo*	S	0–10	47.09

The threshold is defined as the first instance (if any) where more than 30% of the species’ present-day occupancy is lost between habitat loss percentage classes. The species’ mean occupancy decrease is given as a percentage of the present-day occupancy

The mean habitat loss % at which there was a threshold is 40%, with the most frequent being 10%. For *Garrulus glandarius*, *Poecile palustris* and *Bufo bufo* under scenario R1, a threshold amount did not occur until 90% habitat loss. The scenario under which a threshold was most frequently found is R1, but it should be noted that for three species this did not occur until 90% habitat loss.

## Discussion

Understanding the implications of future land cover change on species’ persistence and diversity is crucial, in tandem with an understanding of the social and economic impacts, if a city is to be considered sustainable (Wu 2009). Here we have provided a workflow to compare competing urban landscapes and evaluated the impact of six potential habitat loss propositions on both ecological and social factors. We found that preferentially losing more common habitats and smaller patches tended to be least detrimental for both ecological and social factors.

The method we present allows clear landscape characteristics affecting sustainability to be defined and assessed in a repeatable and transparent way. Quantitative estimates of ecological sustainability can be combined with any criteria important to the project using MCDA, as long as these criteria are quantifiable and measured in consistent units. Caution is needed, however, because these results vary depending on the species and the criteria used in the analysis.

The results of our case study suggest that patch size and irreplaceability are important to consider in urban planning. Irreplaceability is defined as the importance of a site in achieving conservation goals and is related to its rarity (Pressey et al. 1993). Our results are reassuring because they fit with received conservation wisdom and our initial expectations, but our method has the benefit of quantitatively and comparatively estimating the importance of prioritising large and/or rare habitat patches. Judged by all criteria, the highest-ranking future habitat loss scenario in our case study was R1, the scenario where nationally common habitat patches are more vulnerable to loss. Scenario R2, where the locally common habitat patches are lost first is only slightly lower ranking. The distinction between these two scenarios may influence this result. We defined local rarity/commonness through the inclusion of the whole study area. Creating this ranking based on only the city boundary (as some planners might do) could change the results.

When viewing the results by habitat specialism groups, the picture changes somewhat. We found that a land-sparing approach would be likely to benefit generalists and farmland specialists; however, such a configuration would potentially have a negative impact on woodland specialist bird species and amphibian species. The land-sparing versus land-sharing debate comes originally from the agricultural literature (e.g. Green et al. 2005) and refers to an approach where small areas of land are intensively farmed with the rest set aside, versus one where large areas are farmed using wildlife-friendly techniques. In the urban context, land-sharing would be equivalent to sprawling development interspersed with natural areas and land-sparing would be compact cities made up of intensive development but over a smaller area (Lin and Fuller 2013). For generalists and farmland specialists in our case study, scenario groups A1 (smallest patches lost first) and C (developments act as a contagion on the landscape) were rated as highest ranking in our analysis. Because habitat patches within the core of the city tend to be smaller, both of these scenarios have the effect of habitat loss within the core city, and retention in the peri-urban fringe (i.e. land sparing). Conversely, the contagion scenario group C is lowest ranking for the amphibian species, and second lowest ranking (to scenario A1, smallest patches lost first) for woodland bird species. This is to be expected because remnant habitats within cities have been found to offer habitat for species which have been impacted by the intensification of agriculture in the wider landscape (Tratalos et al. 2007).

A configuration such as the ‘compact city’ is also likely to reduce some of the social and educational benefits of green space (Miller and Hobbs 2002), and in the case of our study area potentially fail to meet some of the targets for access to green space such as those outlined by Natural England (2010). This is reflected by the fact that scenario groups A1 and C are the scenarios with the least amount of green space remaining in the city boundary (see Fig. 3). High-density urban developments have previously been found to have a negative impact on the provision of ecosystem services (Tratalos et al. 2007), which is another factor that is important to consider.

The measure of sustainability used to compare scenarios has an important influence on the ranking. In our case study, all three measures of ecological sustainability gave roughly the same ranking: scenario groups A1 and C first, then groups R1 and R2. When judging based on social sustainability alone, groups R1 and R2 were highest ranking. The city green space measure was the only one to rank scenario group A2 highly, and it is also the only measure to not incorporate species’ occupancies. This suggests a potential conflict between social and ecological goals.

We gave equal weight to all criteria, which assumes equal importance of different criteria. It is possible within our method to weight criteria such that some are favoured more highly than others (Kiker et al. 2005). Although weightings are not necessary and can often be subjective (Kiker et al. 2005), weighted MCDA has been found to outperform equal-criteria MCDA in a simulation study (Jia et al. 1998). If weights are used when applying this method, it is important that the weights are appropriate (Wang et al. 2009).

We found that threshold effects were present for all the specialist species studied, and that the difference between planning decisions (i.e. our scenarios) could make the difference between catastrophic population crashes and minimal effects on some species, while for others it makes little difference. Our method can give some indication of which planning decisions may pose this issue, and for which species. The species with the lowest occupancy levels to start with displayed evidence of thresholds for all (*Poecile palustris*, *Bufo bufo*) or most (*Garrulus glandarius*) scenarios. For *Poecile palustris* and *Bufo bufo*, the threshold level of habitat loss was very low (10 and 20% habitat loss) for all scenarios except the two based on habitat type (R1 and R2) which suggests that removal of the rarer habitats can be disastrous for these species. Conversely, the farmland species displayed thresholds under the rarity scenarios with a fairly low percentage of habitat loss (20 and 30%). The existence of habitat loss thresholds is important to consider in land-use planning (Swift and Hannon 2010). The results presented here could add to a growing body of evidence on habitat-loss thresholds after testing their general applicability in other study sites.

The method we present allows clear sustainability criteria to be set up and assessed in a repeatable and transparent way. Quantitative estimates of ecological sustainability can be generated using the IFM, and here we combined them with measures of social sustainability (presence of green space and species within the city boundary). However, any criteria important to the project can be included, as long as they are quantifiable and measured in consistent units. This makes this method of impact assessment particularly useful for urban planning because environmental factors must be considered alongside social and economic factors for a city to be sustainable (Wu 2009). Caution is needed, however, because these results vary depending on the species and the criteria used in the analysis.

Simulating the impact of different habitat scenarios allows us to pick out key characteristics of optimal landscape patterns for species conservation, compare results across different species and criteria, and flag possible thresholds that may lead to catastrophic declines. Our results show that it is not just the amount of habitat that is lost, but also the way it is lost in terms of patch type, size and configuration. The scenarios we developed were simplified versions of the complex dynamics and patterns found in urban landscapes, which may reduce the utility to urban planners of the specific results reported here (Alberti 2005). Our workflow can, however, be applied to any level of complexity in scenario or model design. For example, the IFM could be adapted to incorporate the increased complexity in functional connectivity found in urban landscapes (Tannier et al. 2016) instead of Euclidean distance between patches (e.g. Watts and Handley 2010). The generality of our method should therefore be tested by applying it to other study sites; one of the benefits of the method presented is that as long as data are available, it is fairly easily translated into a new study area.

## Electronic supplementary material

Below is the link to the electronic supplementary material.

Supplementary material 1 (DOCX 17 kb)
