# Supplementary material for: Metapopulation modelling of long-term urban habitat-loss scenarios
Source: Landsc Ecol. 2017 Mar 25;32(5):989–1003. doi: 10.1007/s10980-017-0504-0 (PMC7010366; doi:10.1007/s10980-017-0504-0)
Supplement: Supplementary file 1 — Supplementary material 1 (DOCX 17 kb) [file 10980_2017_504_MOESM1_ESM.docx]

APPENDIX I

**Table S1**. Habitat classes in ascending order of rarity for (a) Great Britain and (b) the study site.

(a)

| **LCM class** | **Area (ha)** | **code** |
| --- | --- | --- |
| Arable and Horticulture | 6251063 | 3 |
| Improved Grassland | 5553921 | 4 |
| Acid Grassland | 1645331 | 8 |
| Coniferous Woodland | 1451100 | 2 |
| Broadleaved Woodland | 1325565 | 1 |
| Heather Grassland | 1323403 | 11 |
| Rough Grassland | 1292015 | 5 |
| Heather | 741079 | 10 |
| Neutral Grassland | 129564 | 6 |
| Fen, Marsh and Swamp | 10006 | 9 |

(b)

| **LCM class** | **Area (ha)** | **code** |
| --- | --- | --- |
| Arable and Horticulture | 3902 | 3 |
| Improved Grassland | 2635 | 4 |
| Broadleaved Woodland | 859 | 1 |
| Rough Grassland | 335 | 5 |
| Neutral Grassland | 196 | 6 |
| Coniferous Woodland | 50 | 2 |
| Heather | 28 | 10 |
| Heather Grassland | 16 | 11 |
| Acid Grassland | 5 | 8 |
| Fen, Marsh and Swamp | 4 | 9 |

**Table S2**. Summary of parameter estimates (means and standard deviations) used in the model simulation. n = 200 for all species. Parameter *x* represents the extent to which a species’ survival is dependent on patch size (larger *x* represents weaker dependence) and parameter *u* is a constant. The critical patch size, below which a species cannot survive in the patch, is given by $u^{\frac{1}{x}}$ (all patches ≤ $u^{\frac{1}{x}}$have extinction probability 1). Parameter *y* represents the level of connectivity required by a species to achieve colonisation.

| **Species** | **Parameter** | **Mean** | **SD** |
| --- | --- | --- | --- |
| *Turdus merula* | x | 0.69 | 0.01 |
| *Turdus merula* | u | 0.07 | 0.00 |
| *Turdus merula* | y | 2895.34 | 31.07 |
| *Prunella modularis* | x | 0.77 | 0.01 |
| *Prunella modularis* | u | 0.05 | 0.00 |
| *Prunella modularis* | y | 2524.30 | 46.21 |
| *Carduelis chloris* | x | 0.92 | 0.05 |
| *Carduelis chloris* | u | 0.55 | 0.06 |
| *Carduelis chloris* | y | 1566.09 | 104.05 |
| *Emberiza calandra* | x | 1.78 | 0.07 |
| *Emberiza calandra* | u | 12.91 | 1.79 |
| *Emberiza calandra* | y | 4852.17 | 586.92 |
| *Passer montanus* | x | 0.91 | 0.03 |
| *Passer montanus* | u | 0.54 | 0.18 |
| *Passer montanus* | y | 4936.92 | 882.12 |
| *Emberiza citrinella* | x | 0.45 | 0.02 |
| *Emberiza citrinella* | u | 0.23 | 0.02 |
| *Emberiza citrinella* | y | 2485.04 | 130.12 |
| *Garrulus glandarius* | x | 0.69 | 0.04 |
| *Garrulus glandarius* | u | 0.63 | 0.03 |
| *Garrulus glandarius* | y | 105.05 | 4.52 |
| *Poecile palustris* | x | 1.92 | 0.09 |
| *Poecile palustris* | u | 8.50 | 0.83 |
| *Poecile palustris* | y | 28.24 | 2.05 |
| *Rana temporaria* | x | 0.50 | 0.00 |
| *Rana temporaria* | u | 0.14 | 0.00 |
| *Rana temporaria* | y | 213.62 | 1.44 |
| *Bufo bufo* | x | 1.10 | 0.05 |
| *Bufo bufo* | u | 0.03 | 0.01 |
| *Bufo bufo* | y | 229.20 | 40.23 |
